# A Comparative Cross-Sectional Study of the Consequences of the COVID-19 Lockdown on Women’s Health Behaviors in Spain

**DOI:** 10.3390/nu14040846

**Published:** 2022-02-17

**Authors:** María José González-Calderón, José I. Baile, Eva Izquierdo-Sotorrío

**Affiliations:** Department of Psychology, Faculty of Health Science and Education, Madrid Open University, 28400 Collado-Villalba, Spain; mariajose.gonzalez@udima.es (M.J.G.-C.); eva.izquierdo@udima.es (E.I.-S.)

**Keywords:** COVID-19, lockdown, health habits, body weight, women’s health

## Abstract

Changes in health habits were observed during the COVID-19 pandemic. An unequal distribution of responsibilities may have generated unequal levels of stress in men and women, and, therefore, this may have led to different impacts on their health habits during lockdown. This study aimed to analyze the changes in eating habits, physical activity, sleep, and body weight in women, compared to men, because of lockdown. A comparative cross-sectional study was carried out. 2834 Spanish volunteers aged 19 to 76 completed an online survey on health habits. Descriptive and inferential analyses were performed using a cross-sectional methodology to explore gender differences. Results showed that men maintained their health habits to a greater extent, performing the same physical activity, while maintaining the quantity and quality of sleep, the quality of the diet, the quantity of the intake, and their mealtimes and body weight, whereas women’s eating habits changed in relation to the quality of their diet and mealtimes, as their food intake and weight increased, and their sleep was poorer in quality and duration than it was before lockdown. This could be due to the higher levels of anxiety experienced by women as a result of working on essential jobs in addition to taking on more unpaid work associated with care and housework.

## 1. Introduction

The COVID-19 pandemic has caused an unprecedented health and economic crisis on a global scale. Spain was among the countries most affected in the early stages of this crisis. For two months, non-essential services were interrupted, and “stay-at-home” measures were adopted in order to limit people’s movement. The stressors associated with the lockdown include its prolonged duration, fear of the virus itself, lack of information, economic or work-related concerns, and uncertainty about the future [1,2]. Women were also impacted by the unequal distribution of responsibilities related to child and elderly care, and to household chores among heterosexual couples [3].

Pandemics and outbreaks have differential impacts on women and men. From risk of exposure to the social and economic implications, individuals’ experiences are likely to vary according to their biological and gender characteristics, and to their interactions with other social determinants. However, data on gender differentials pertaining to the impact and consequences of changes in health habits during the COVID-19 lockdown are still limited, even though the disaggregation of data by gender is a repeated recommendation of international organizations and researchers who study the determinants of health (both physical and psychological) [4,5]. This disaggregated approach seeks to understand, among other issues, the possible psychosocial factors that create specific gendered risks to health, with the aim of improving health promotion policies and health risk prevention strategies [6]. In this respect, the World Health Organization (WHO) encourages the Member States and their partners to collect, report, and analyze data on COVID-19 that are disaggregated by sex and age, and, additionally, the WHO urges them to conduct a gender analysis of data while investing in quality gender-responsive research on the potentially differential adverse health, social, and economic impacts of COVID-19 on women and men [7].

Since the COVID-19 lockdown, which started in 2020, changes in health habits have been observed in a range of populations; these include changes in eating habits [8,9], increases in food consumption [10,11], consequent increases in body weight [9,12], reductions in physical activity [13], and a worsening of sleep quality [14]. However, few studies have analyzed the impact of lockdown on these habits relative to gender. The COVID-19 crisis led to the closure of schools, nurseries, and other childcare facilities, significantly changing the “care economy” while transferring to households both the care and education of children as well as the care of the elderly, sick, and other dependents; these are tasks traditionally performed by women [15]. This led to a considerable increase in the daily time devoted by women to unpaid care work [16]. Research suggests that women undertook three times more unpaid care work than men during the pandemic, while caring for family members infected with COVID-19 further increased this burden [17]. These trends have the potential to generate unequal levels of stress in men and women, and, consequently, this causes different impacts on their health habits.

To expand the knowledge on the differential impacts of lockdown on men and women, and, following the recommendations of the World Health Organization in relation to the research on COVID-19 pandemic, the aim of the present study is to analyze the changes in the health habits of women in comparison to men, as a consequence of the strict lockdown enacted in Spain in response to the pandemic. In particular, the changes in eating habits, physical activity, and sleep, as well as the impact of these changes on body weight, are analyzed. It is hypothesized that the consequences of lockdown measures, pertaining to health habits, differ by gender, causing a greater impact on Spanish women due to higher levels of anxiety resulting from the intensification of labor distribution because of traditional gender roles and larger changes in the care economy.

## 2. Materials and Methods

### 2.1. Participants

A convenience sample occurred in this study. It consisted of 2834 Spanish participants aged between 19 and 76 (M: 41.36; SD: 10.5), 69.3% of whom were women. 32.5% were aged 19 to 35, 44.7% were aged 36 to 49, and 22.8% were aged 50 or older. A total of 86.7% held university degrees or had attended college. During the lockdown period, 81.4% worked outside the home, performing a job considered essential according to the Spanish Government guidelines. The participants were members of the Madrid Open University community (students, professors, or administration staff) who were asked to participate in the study anonymously on a voluntary basis. They were not asked to provide any identification information. The inclusion criteria were: (1) to be 18 years or older; (2) to complete the form in full; and (3) to send informed consent to participate in the study.

### 2.2. Survey Design and Administration

After receiving authorization to proceed with this research from the Ethics Committee of the Faculty of Health Sciences and Education at Madrid Open University, the link to an online survey was sent to the university community by email. The 20-item self-report survey was developed specifically for the present study to collect the following data on the participants: (a) sociodemographic data (sex, age, height, weight, educational level, having suffered COVID-19 during the lockdown, and having lived in the same place as someone who suffered it); (b) information about their weight assessed through a scale (changes in weight in number of kilos gained/lost); (c) changes in their eating habits (general eating habits, the quality and variety of the diet, the amount of food ingested, meal times, and the effect of anxiety on eating habits); (d) changes in their physical activity (frequency and number of hours a day); and (e) changes in their sleeping habits (quality and quantity of sleep) and average sleeping hours.

It was expressly stated that participation was voluntary, and that the information collected would be confidential and used for research purposes only, in accordance with Spanish Law (3/2018) on the Protection of Personal Data and Guarantee of Digital Rights. Responses were coded anonymously using individual identification numbers. Only those who provided informed consent on the first page of the online form were able to complete the Google Forms Survey. Besides, answering a question was required in order to move to the next one, and only complete forms were recorded in the database. Anonymity, together with the fact that no participants were paid or received any academic benefit for their participation, aimed to address potential sources of bias. As for the response rate (31.1%), it can be considered high because the Spanish population was going through the stresses of the pandemic. The period of participation was 19 May to 31 May 2020, following the period of strict lockdown in Spain, which took place from 14 March to 10 May. In order to avoid duplication of subjects, participants were only able to complete the survey once. They were given 15 min to do so.

### 2.3. Data Analysis

Descriptive and inferential analyses were performed using a cross-sectional methodology that provided a snapshot of the changes in different health habits of Spanish men and women, at a single point in time, just after the lockdown finished. Percentages were calculated to determine the distribution of sociodemographic variables and health habits during lockdown. The chi-square test was used to identify relationships between variables, while Cramer’s V was used to determine the power of the relationships found. Data were coded and analyzed by means of the SPSS software package, version 21.0 for Windows. There were no missing data.

## 3. Results

### Descriptive Analysis of the Participant Sample

The height and weight of the participants were self-measured, and Body Mass Index (BMI) was calculated. According to their BMI, participants were grouped into these four categories following the cut-off points set by the World Health Organization: underweight (below 18.5), normal-weight (18.5–24.9), overweight (25–29.9), and obesity (above 30). In this sample, the largest weight group was “normal weight”, followed by “overweight”; the underweight and obese groups were notably smaller. Statistically significant gender differences were found, as men were more likely to be overweight or obese, while a higher proportion of women were classified within the normal weight group (Table 1).

The analyses showed that there were no significant gender differences in sociodemographic variables, such as level of education (Table 2). There were also no significant gender differences in the percentage of participants who suffered COVID-19 or lived in the same household as someone who suffered it. However, during the period of strict lockdown in Spain, women worked outside the home at a statistically significant higher rate than men (84.9% vs. 73.3%). As a consequence of this, in addition to carrying out subsequent analyses using data from the total sample, subsamples of participants, based on whether they had worked outside the home during lockdown, were also analyzed. However, similar results were obtained when analyzing the total sample and the two subsamples separately, and thus only the results of the total sample are presented below.

Lockdown resulted in a significantly different influence on the eating habits of male and female participants. When asked about the changes in their eating habits, men were more likely than women to report that their habits had remained mostly unchanged during lockdown. Conversely, a higher percentage of women expressed that their eating habits had changed; they were more likely to describe the change as improvement rather than deterioration (Table 3). Similar results were found in relation to the quality and nutritional variety of participants’ diets: men were more likely to have stuck to their pre-lockdown diets, while more women expressed that their diets had improved or worsened.

Results were similar with regard to the amount of food consumed, as a greater proportion of men stated that their consumption remained the same, or even decreased, while more women said that their food intake had increased. Likewise, a higher percentage of men stated that their mealtimes had not been affected, while a higher proportion of women reported that their mealtimes had undergone changes during the period under study. Those subjects who regularly weighed themselves using a scale at home were asked to provide their body weight. Statistically significant differences were found in responses between men and women, with more women stating that their weight had increased, and more men reporting that their weight had decreased. However, roughly equal numbers of women and men reported that their weight had remained the same. Further gender differences appear in degrees of weight change experienced. Men presented higher percentages of weight loss, which are more pronounced in the ranges of 2–5 kg and more than 5 kg. Women, on the other hand, presented higher percentages of weight gain in the range of 0–2 kg. It should be noted that no participant reported weight gains higher than five kilos. Finally, women were significantly more likely than men to experience nervousness or anxiety during the lockdown (68.1% vs. 52.9%). Furthermore, among those who reported experiencing these symptoms, women were more likely than men to report that this symptomatology caused a negative effect on their eating habits (including amount of food eaten, number of meals per day, quality of food consumed, and way of eating, etc.)

No statistically significant gender differences were observed in weekly frequency of exercise or daily hours dedicated to physical activity during lockdown (Table 4). However, significant differences were observed in the participants’ subjective perceptions of exercise during lockdown compared to that prior to lockdown. While men and women exercised at similar rates during lockdown, men were more likely to say that this level of activity was similar to or less than their pre-lockdown level, while women were more likely to say that their activity levels had increased.

The results show that both the quantity and quality of participants’ sleep were affected during the lockdown, and that this effect was significantly different in men and women (Table 4). Regarding the quality of sleep, a higher percentage of men reported that they had slept as much as usual during lockdown, while women more often reported that they had slept worse, or much worse, than usual. Men were also more likely to report that they had slept the same number of hours each night as in the period prior to the lockdown, while a higher proportion of women indicated that they had slept either fewer hours or more hours than before.

## 4. Discussion

The results of the present study indicate that men maintained their health habits during the lockdown to a greater extent than women, performing the same physical activities while maintaining the same quantity and quality of sleep. Men also maintained similar eating habits, including quality of diet, quantity of food intake, and mealtimes. This may explain the fact that men were more likely than women to have maintained or reduced their body weight in that period.

A significant proportion of the families confined during the COVID-19 pandemic developed domestic work patterns echoing traditional structures that link women to the domestic sphere and assign them, as a priority, work related to care and household chores [3], which means that women took on more unpaid work during the lockdown than men. The women of this sample were also significantly more likely than men to work outside the home, as they performed more of the jobs considered essential during the lockdown. This circumstance, in an already complex situation, together with the pre-established unequal sharing of caring tasks in the Spanish population [18], could have contributed to the higher percentages of anxiety detected in women, which, in turn, could explain the changes detected in their health habits. The higher levels of anxiety experienced by women are in line with the results of studies conducted during the pandemic [19,20], and these findings also agree with previous scientific evidence suggesting that women tend to be more prone to experiencing stress and developing post-traumatic symptoms [21].

These findings, which indicate that a significantly higher proportion of women experienced nervousness or anxiety during lockdown, worsening their eating habits, are in line with studies that describe food as “emotional nourishment”; these studies identify food intake as a response to emotions such as anxiety, boredom, or stress [22], all of which were present during lockdown. Therefore, the findings suggest the existence of a gender-specific response to stress [23], with women being more likely to use food intake to manage stress. Furthermore, changes in eating habits, especially increased food intake and particularly the intake of calorie-rich foods, as well as the greater weight gain experienced by a higher proportion of women than men during lockdown, have also been highlighted in other studies focused on this period [8,12,24]. However, in some research, no significant differences were found by gender with respect to the increase in food intake during quarantine [11].

The reported increase in physical exercise in a higher proportion of women, and the decrease in a higher percentage of men, compared to activity patterns prior to lockdown, is consistent with similar studies [25], although other studies have not found gender differences [26,27]. This result could be explained by the greater predisposition of women to exercise at home [27], while men tend to practice physical activity mainly for social and competitive reasons, and they are more likely to do so in public places [28].

As in similar studies [14], the results indicated a worsening of sleep quality, at a higher percentage, for women when compared to men. This may be related to the higher levels of anxiety experienced by women, and to alteration of daily routines (e.g., mealtimes, exercise) at a higher rate than that reported among men. All these factors can affect sleep-wake cycles, which have been observed to be disrupted during the lockdown [29].

Despite the possible biases of social desirability, and the bias linked to the recall of health habits, the self-perceived state of health remains a useful indicator when assessing the lifestyle of the population. To solve these biases, on the one hand anonymous participation was requested, and on the other hand objective inputs were used, such as body weight measured on scales. Likewise, since it is a convenience sample made up of people with a high educational level, the results cannot be generalized to the whole of Spain’s population. However, the study sample appears to be representative, as the recorded Body Mass Index (BMI) distribution is very similar to that obtained in previous studies of Spain’s population [30,31]. Surveys such as EUROSTAT [32] have systematically recorded differences in the self-perception of men and women, with women being slightly less optimistic than men in terms of their habits, although these differences have never been particularly significant in the Spanish population, and thus do not account for the significant differences observed in the present study. Finally, it is important to note that the survey was developed specifically for the present study, during the pandemic period, and it was to be used immediately after the strict lockdown finished, so there was no time to validate it.

## 5. Conclusions

Considering that the COVID-19 pandemic is still ongoing, additional longitudinal research is needed, with large and representative samples that cover the entire territory of Spain and employ additional objective measures of women’s health habits. It is important to promote knowledge of how gender and psychosocial variables interact with health habits in order to develop prevention programs that favor equity, providing an equal distribution of resources adjusted to the needs of women and men.

## Figures and Tables

**Table 1:** Sample distribution according to sex and Body Mass Index (BMI).

Sex	*n* (%)	BMI				*p*	*Cramer’s V*
		Underweight	Normal Weight	Overweight	Obesity		
Men	846 (30.8%)	8 (0.9%)	335 (39.6%)	390 (46.1%)	113 (13.4%)	<0.001	0.316
Women	1902 (69.2%)	99 (5.2%)	1289 (67.8%)	380 (20%)	134 (7.0%)		
Total	2748 (100%)	107 (3.9%)	1624 (59.1%)	770 (28%)	247 (9%)		

**Table 2:** Gender differences in sociodemographic variables.

Sociodemographic Variables	Women	Men	*X*^2^ (*gl*)	*p*	*Cramer’s V*
	(*n* = 1964)	(*n* = 870)			
**Age**					
≤35 years	36.60%	23.10%	93,517 (2)	<0.001	0.182
36–49 years	45.20%	43.70%			
≥50 years	18.20%	33.20%			
**Educational level**					
Primary	0.20%	0.10%	3162 (3)	0.367	**ns** ^1^
Secondary	4.60%	6.20%			
Vocational training	8.10%	7.80%			
Higher education	87.10%	85.90%			
**COVID-19 patient**					
Yes	10.70%	10.10%	0.214 (1)	0.644	**ns**
No	89.30%	89.90%			
**Cohabiting COVID-19 patient**					
Yes	9.50%	10.80%	1205 (1)	0.272	**ns**
No	90.50%	89.20%			
**Work outside home**					
Yes	84.90%	73.30%	53,473 (1)	<0.001	0.137
No	15.10%	26.70%			

^1^ Note: ns (non-significant).

**Table 3:** Changes in eating habits and weight during confinement.

*n* = 2834	Women	Men	*X*^2^ (*gl*)	*p*	*Cramer’s V*
	(*n* = 1964)	(*n* = 870)			
**Eating Habits**					
Maintained	39.10%	46.80%	16,003 (2)	<0.001	0.075
Improved	33.00%	30.50%			
Worsened	27.90%	22.80%			
**Diet quality and variety**					
Maintained	43.20%	51.10%	15,656 (2)	<0.001	0.074
Improved	37.60%	31.70%			
Worsened	19.20%	17.10%			
**Intake amount**					0.077
The same	38.30%	41.40%	16,907 (2)	<0.001	
Increased	46.10%	38.50%			
Reduced	15.50%	20.10%			
**Schedule change**					0.081
Yes	39.60%	31.00%	18,821 (1)	<0.001	
No	60.40%	69.00%			
**Weight (scale)**					
Maintained	36.60%	36%	15,222 (2)	<0.001	0.086
Increased	42.20%	35.40%			
Reduced	21.20%	28.60%			
**Kilos (gained/lost)**					
Lost (≥5 kg)	4.60%	7.10%	27,726 (5)	<0.001	0.116
Lost (2–5 kg)	6.50%	11.50%			
Lost (0–2 kg)	13.30%	14.70%			
Maintained	35.60%	34.50%			
Gained (0–2 kg)	23.90%	17.70%			
Gained (2–5 kg)	16.10%	14.40%			
**Anxiety**					
Yes	68.10%	52.90%	60,479 (1)	<0.001	0.146
No	31.90%	47.10%			
**Effect of anxiety on eating habits**	**(*n* = 1467)**	**(*n* = 573)**			
No effect	43.80%	51.30%	14,438 (2)	<0.001	0.084
Worsening	48.40%	39.10%			
Improvement	7.80%	9.60%			

**Table 4:** Change in sleep and physical activity habits during confinement.

*n* = 2834	Women	Men	*X*^2^ (*gl*)	*p*	*Cramer’s V*
	(*n* = 1964)	(*n* = 870)			
**Frequency of physical activity**					
No physical activity	25.10%	25.90%	7010 (4)	0.135	**ns** ^1^
1–2 days a week	23.50%	26%			
3–4 days a week	21.10%	17.70%			
5 days a week	17.20%	15.70%			
Everyday	13.10%	14.70%			
**Physical activity (hours per day)**					
No physical activity	27.30%	28.70%	1018 (3)	0.797	**ns**
<1 h/day	42.80%	41.70%			
1–2 h/day	29.30%	28.70%			
≥3 h/day	0.60%	0.80%			
**More or less physical activity than before**					
Considerably less	35.70%	39.30%	43,967 (4)	<0.001	0.125
Something less	21.30%	24.30%			
The same	11.80%	16.00%			
Something more	21.50%	16.20%			
A lot more	9.80%	4.30%			
**Sleep quality**					
As always	40.50%	52.10%	37,272 (2)	<0.001	0.115
Worse	40.30%	35.40%			
A lot worse	19.10%	12.50%			
**Sleeping hours**					
The same	33.50%	42.60%	21,799 (2)	<0.001	0.088
Less hours	32.70%	28.40%			
More hours	33.80%	29.00%			
**Average sleeping hours**					
Less than 6 h	15.80%	16.10%	9952 (3)	0.019	0.059
6–7 h	38.60%	42.10%			
7–8 h	35.10%	34.90%			
More than 8 h	10.40%	6.90%			

^1^ Note: ns (non-significant).

## Data Availability

The data presented in this study are openly available in OSF Registries at https://doi.org/10.17605/OSF.IO/HJCDQ, accessed on 17 August 2021.
